# The Satiating Properties of Pork are not Affected by Cooking Methods, Sousvide Holding Time or Mincing in Healthy Men—A Randomized Cross-Over Meal Test Study

**DOI:** 10.3390/nu9090941

**Published:** 2017-08-26

**Authors:** Ursula Kehlet, Bhaskar Mitra, Jorge Ruiz Carrascal, Anne Raben, Margit D. Aaslyng

**Affiliations:** 1 Danish Meat Research Institute (DMRI), Gregersensvej 9, DK-2630 Taastrup, Denmark; mdag@teknologisk.dk; 2 Department of Food Science, Faculty of Science, University of Copenhagen, Rolighedsvej 30, DK-1958 Frederiksberg C, Denmark; bhaskar@food.ku.dk (B.M.); jorgeruiz@food.ku.dk (J.R.C.); 3 Department of Nutrition, Exercise and Sports, Faculty of Science, University of Copenhagen, Rolighedsvej 26, DK-1958 Frederiksberg C, Denmark; ara@nexs.ku.dk

**Keywords:** meat proteins, appetite, satiety, in vitro digestion, protein digestibility, pork, sous-vide, low temperature long time, cooking

## Abstract

Low temperature long time (LTLT) sous-vide cooking may modify meat proteins in a way that could promote satiety. We investigated the effects of (1) cooking method (LTLT 58 °C vs. oven 160 °C), (2) LTLT holding time (17 h vs. 72 min), and (3) pork structure, LTLT 58 °C for 17 h (minced vs. roast) on appetite regulation and in vitro protein digestibility. In a cross-over study, 37 healthy men consumed four meals containing pork: LTLT-cooked roast, 58 °C, 72 min; LTLT-cooked roast, 58 °C, 17 h; and, oven-cooked roast, 160 °C to a core temperature of 58 °C and LTLT-cooked minced patties, 58 °C, 17 h. Ad libitum energy intake (EI) after three hours was the primary endpoint. Moreover, subjective appetite sensations were assessed. Protein digestibility was determined in an in vitro simulated digestion model. Ad libitum EI did not differ between the meals. Furthermore, appetite ratings were not clearly affected. LTLT cooking for 72 min increased the proteolytic rate in the early gastric phase during digestion as compared to LTLT cooking for 17 h or oven cooking. In conclusion, LTLT cooking, LTLT holding time, and pork structure did not affect ad libitum EI. However, LTLT cooking at 58 °C for 72 min seemed to enhance in vitro protein digestibility.

## 1. Introduction

Foods that have the capacity to reduce hunger and further food intake are important in the fight against obesity and its related diseases. Thus, appetite is regulated by a complex interplay of psychological factors and physiological responses related to the macronutrient composition, energy density, sensory quality, and physical structure of foods [1]. Dietary proteins have been shown to be one of the most satiating macronutrients [2], and they appear to increase thermogenesis [3], stimulate appetite-regulating hormones [4], and increase the concentration of circulating amino acids [5]. Also, the rate of protein digestion and absorption is proposed as one of the underlying mechanisms of protein-induced satiety, since it has been shown that rapidly absorbed proteins are more satiating than slowly absorbed proteins [6,7]. The satiating effects of proteins could therefore be variable and depend on the characteristics of the specific protein. Proteins differ in their amino acid composition, structure, and digestibility, and proteins from animal sources generally have a higher digestibility than proteins from plant sources [8,9].

Meat is an important source of dietary proteins and normally undergoes heat treatment prior to ingestion. Cooking meat at low temperatures for prolonged times is widely used in the food service industry and also recently in home cooking due to the possibility of achieving a consistent and appealing eating quality [10]. The method is generally termed sous-vide, meaning that the meat is vacuum-packed and cooked in a water bath at temperatures below 100 °C. A special variant of sous-vide is cooking at a low temperature for a long time (LTLT) [11,12]. In LTLT heat treatment, the meat is heated at temperatures between 55 °C and 65 °C for several hours. LTLT holding time is defined as the time the meat is cooked in the water bath after a specific core temperature has been reached.

Heating may induce structural modifications to meat proteins [13] that could improve (by denaturation) or reduce (by aggregation, oxidation) the protein digestion rate. Since the latter in turn influences the postprandial amino acid response [14], the cooking procedure could play a role in the satiating effect of meat proteins. In porcine muscles, LTLT cooking at 58 °C for a prolonged time (holding time of 20 h) results in actin denaturation [15], even though actin has previously been reported to denature at approximately 77 °C [16]. Myosin seems to be fully denatured already at a cooking temperature of 53 °C, after a holding time of three hours [15]. The denaturation of actin and myosin could increase the accessibility of cleavage sites to proteolytic enzymes [17]. Moreover, the proteolytic enzyme system, cathepsins B + L, has been found to remain active after 24 h cooking at 58 °C [18], potentially leading to a greater release of free amino acid during cooking, which is more rapidly absorbed during the subsequent digestion. On the other hand, meat proteins undergo structural changes during cooking and possibly for prolonged cooking times that lead to protein-protein interaction, resulting in aggregation [13]. Thus, the cooking temperature may lead to counteracting effects on the subsequent uptake of amino acids during digestion. Bax et al. [17] concluded that the cooking temperature of meat is a determinant of the protein digestion rate. Porcine *Longissimus dorsi* cooked in a 70 °C water bath resulted in a faster digestion of meat proteins than cooking temperatures of 100 °C or above. However, these in vitro digestion experiments were performed on extracted myofibrillar proteins and need to be confirmed in the original matrix of the proteins (meat) and under controlled heating conditions with measurements of core temperatures during cooking. Mincing is a common process in the meat industry and may affect the physical and chemical properties of the meat proteins. Minced beef has been shown to increase protein digestion and absorption in vivo as compared to whole muscle [19]. Thus, minced meat is expected to facilitate a faster satiety response than whole muscle. 

Based on the expected differences in protein digestibility, we hypothesized that the ingestion of LTLT-cooked pork (58 °C) would induce a faster satiety response and subsequently a lower energy intake (EI) as compared to oven-cooked pork (160 °C). Moreover, LTLT-cooked pork cooked for 17 h would be more satiating than pork cooked with a holding time of 72 min. Finally, we hypothesized that minced pork would be more satiating than pork roast. 

## 2. Materials and Methods 

### 2.1. Study Design

A semi-controlled cross-over meal test study was conducted to investigate the effects of (1) heat treatment (LTLT sous-vide at 58 °C compared to oven at 160 °C), (2) LTLT holding time (17 h compared to 72 min), and (3) pork structure (minced pork compared to roast) of pork *M. Semitendinosus* on appetite regulation (Table 1). The study was carried out at the Danish Meat Research Institute (DMRI) in Denmark from November to December 2014. The test meals were served as lunch meals, and an ad libitum meal was served three hours later. Prior to the test meal, a standardized breakfast was served to the participants at the study site. The semi-controlled conditions allowed the participants to continue their daily lives between the meals, provided it only included sedentary physical activity. However, the participants were given careful instructions not to consume any foods or drinks between the breakfast and test meal, and between the test meal and the ad libitum meal. However, between the meals (breakfast and test meal, and test meal and ad libitum meal), the participants were provided with a bottle of water (0.25 L) from which they were allowed to drink. They returned the bottles, and the amount of consumed water was calculated. During the study period, the participants were instructed to otherwise maintain their ordinary lifestyle.

Each test meal was separated by at least a two-day wash-out period. Prior to each study day, the participants were instructed to fast from 10 pm the evening before, but were allowed to drink 0.5 L of water until the next morning. Furthermore, the participants were instructed to abstain from alcohol and hard physical activity for 24 h prior to each study day. On each study day, the participants were served a standardized breakfast upon arrival. The breakfast meal consisted of a wheat bun (62 g), jam (20 g), cheese (20 g), yoghurt (150 g), and an optional drink of water, coffee or tea (156 mL). On the first study day, the participants chose their drink, which was noted, and the same drink was served on the remaining study days. The breakfast meal had an energy content of 2 MJ (15% of energy (E%) from protein, 55 E% from carbohydrates and 30 E% from fat). 

The test meals were served four hours after the standardized breakfast. Subjective appetite sensations were assessed by using visual analogue scales (VAS) prior to the test meal. The participants were instructed to consume the test meal within 15 min. After the test meal had been consumed, the participants rated the palatability of the test meal by using VAS. VAS questionnaires on appetite were completed every 30 min: at time points 15, 45, 75, 105, 135, 165, 195 min from the termination of the test meal. Three hours after the test meal had been consumed, an ad libitum meal was served (time point 195 min). The ad libitum meal consisted of pasta Bolognese (539 kJ/100 g; 23 E% from protein, 48 E% from carbohydrates and 29 E% from fat). The participants were instructed to eat at a constant pace until they felt comfortably satiated. Ad libitum EI was calculated from the amount of food consumed. The test meal and ad libitum meal were consumed in individual booths in the sensory laboratory at DMRI. 

### 2.2. Study Participants

Recruitment was carried out at a Danish workplace (Danish Technological Institute, Taastrup, Denmark) through posters and the in-house intranet. The inclusion criteria were as follows: men, 18–55 years of age, body mass index (BMI) 18.5–27.5 kg/m^2^, pork eaters, and the provision of written consent to participate in the study. Participants were excluded if they: had any food allergies, dislikes or special diets of relevance to the study; regularly used prescription medicine and dietary supplements including protein supplements, shakes and powders; had irregular eating schedules; were on a weight-loss diet or had experienced a weight change (±3 kg) in the previous three months; had known chronic diseases; were vigorously physically active > 10 h/week; were smokers; worked in appetite or related research fields; participated in other clinical trials; and, were unable to comply with the study protocol. 

Eighty potential participants were interested in participating in the study, 43 of whom attended information meetings and underwent screening procedures. Subsequently, 40 participants were deemed eligible and enrolled in the study. All of the participants who fulfilled the inclusion and exclusion criteria gave their written consent to participate in the study after they had received verbal and written information about the study. The participants were randomly assigned to a sequence of four test meals by the study coordinator using a balanced block design. The study was registered at clinicaltrials.gov as NCT02495870, and was carried out in accordance with the Helsinki II Declaration. The Danish National Committee on Health Research Ethics was notified about the study protocol; however, no ethical approval was required, since no biological material was taken.

### 2.3. Test Meals

The four test meals were designed to be isocaloric and identical in their macronutrient composition based on a pilot study measuring the cooking loss of the pork (28% cooking loss for pork patties). However, the actual cooking loss of the pork appeared to differ from the results obtained from the pilot study. This resulted in small differences in the actual protein content and energy density of the meals (Table 1). Due to the study design, it was not possible to adjust for protein or energy content in the statistical analyses, and therefore a separate analysis was performed for cooking loss to investigate differences between the meals.

Meat (porcine *M. Semitendinosus*) was obtained from Duroc (sire), Landrace-Yorkshire (sow) (DLY)-crossbred female pigs (“Antonius special pigs”) that were slaughtered on the same day (slaughter weight: 75–95 kg). Forty-eight hours *post mortem*, the pH was measured in duplicate in each muscle. Muscles with a mean pH of 5.58–5.79 were selected for the appetite study (mean pH of the selected muscles was 5.65 ± 0.05). Mean weight of the muscles were 344.3 ± 52.6 g. The muscles were randomly assigned to each of the four cooking conditions according to the test meals described in Table 1. There was no difference in the pH of the muscles between the four cooking conditions (*p* = 0.27). A holding time of 17 h was chosen based on previous results showing that LTLT cooking at 58 °C for 17 h increased the proteolytic activity from endogenous cathepsins B + L as compared to 5 h or 0 min holding time [11]. A holding time of 72 min was chosen as this is the minimum time to achieve the desired elimination of bacteria during LTLT cooking at 58 °C according to DMRI’s guidelines. 

LTLT cooking was performed for whole muscles (roasts) and for minced pork patties. For the preparation of pork patties, the muscles were minced one time through a 3 mm perforated disc and formed into patties using a circular meat-pattie mould (140 g, diameter: 10 cm, height: 1.5 cm). Prior to LTLT cooking, the whole muscles (one muscle per bag) and pork patties (five patties per bag) were vacuum-packed (VM 51/2, Röscher Vakuumtechnik, BersenBrück, Germany). LTLT cooking was performed in a sous-vide water bath (40 kg Sousvide, Classic Gastro A/S, Marslev, Denmark) at 58 °C for a holding time of 72 min or 17 h. The core temperature was monitored continuously during cooking using temperature data loggers (Testo T175-T2, Buhl & Bønsøe, Smørum, Denmark) inserted lengthwise into a selected “dummy” meat sample (only used for this purpose) for each cooking method. After the desired holding times had been reached, the cooking process was stopped by placing the bags containing the cooked meat in ice water for two hours. Oven cooking was carried out on trays in a pre-heated (160 °C) convection oven (Electrolux air-O-steam 2005, Hvidovre, Denmark) until a core temperature of 58 °C was reached. The cooked roasts were subsequently cooled for 10 min at room temperature and then vacuum-packed. All of the cooked muscles were stored in vacuum bags in a refrigerated room at 0 °C until further use.

On each study day, the vacuum-packed roasts and the meat patties were re-heated in a pre-heated water bath (58 °C) until a core temperature of 58 °C was reached. The temperature was controlled using a cooking thermometer (Testo 926, Buch & Holm, Smørum, Denmark) inserted lengthwise into one of the meat samples for each cooking method (approximate heating times were 90 and 60 min. for the roasts and pork patties, respectively). Prior to serving, the muscles were removed from the vacuum bags and pan-fried for 1 min on each side at 220 °C (4 g butter per muscle or two meat patties). After pan-frying, the whole muscles were cut into slices of pork (15 mm thick). For each participant, approximately 4.5 slices of pork was served providing 202 g of pork per meal. 

Raw and cooked weights of the meat were recorded in order to calculate the cooking loss percentage:

Cooking loss = (weight of raw meat – weight of cooked meat) × 100/weight of raw meat(1)

### 2.4. Visual Analogue Scales

Palatability and sensations of hunger, satiety, fullness, and prospective food intake were measured using VAS. The VASs consisted of a 100 mm horizontal unbroken line with words anchored at each end describing the extremes, e.g., for hunger: “I am not hungry at all”–“I have never been hungrier” [20], and for palatability: “Not at all like”–“Like a lot”. The participants were instructed to place a vertical mark through the horizontal line corresponding to their perceived feeling at that particular time. The intensity of the feeling (distance of the vertical mark from the origin on the left) was measured, thus yielding a score in the range of 0–100 mm. On the first study day, the participants chose whether to assess the VASs using an electronic-based VAS (eVAS) or using the traditional pen and paper method. This was done for the convenience of the study participants, and they were instructed to use the same assessment method throughout the entire study. An electronic-based VAS questionnaire has been shown to be comparable to the traditional pen and paper method [21]. The eVAS system was set up using a web-based questionnaire (Eye question®, Elst, The Netherlands). At the study site, the participants filled out eVASs using desktop computers located in the sensory booths in the sensory laboratory. Between the test meal and the ad libitum meal, the eVAS questionnaires were sent to the participants by email, and the time at which each questionnaire was completed was recorded. The four appetite ratings were combined into a composite satiety score (CSS), which was calculated for each time point by using the following equation [22]:

CSS = (satiety + fullness + (100 − prospective food intake) + (100 − hunger))/4(2)

### 2.5. Warner Bratzler Shear Force

Texture analyses were only performed on whole muscles (the roasts). Meat samples were taken from cooked muscles (five muscles per cooking method) by drilling with a hollow bit into the meat sample to extract a cylindrical sample parallel to the orientation of the muscle fibers (1.3 cm in diameter and 4.5 cm in length). For each muscle, eight samples were extracted. The samples were stored at 5 °C until analysis. The maximum shear force (Warner Bratzler shear force) was measured using a TA-HDi Texture Analyzer (Stable Micro Systems, Goldalming, UK), equipped with a triangular Warner Bratzler test cell (Pre-test speed: 3.5 mm/s, test speed 3.5 mm/s and post-test speed: 10.0 mm/s). 

### 2.6. In Vitro Protein Digestibility

The protocol for in vitro protein digestibility studies was adapted from the international consensus paper for in vitro digestion methods [23], as outlined below with modifications in terms of sample preparation and measuring proteolytic activity at 280 nm. The in vitro digestion protocol mimicked chewing behavior following two phases of digestion: (1) gastric conditions, and (2) duodenal conditions.

#### 2.6.1. Chemicals

For gastric digestion, gastric pepsin (porcine gastric mucosa, P6887) was used. For duodenal digestion, bile extract porcine (B8631), pancreatic trypsin (porcine pancreas, 1000–2000 U/mg, T7409), and α-chymotrypsin (bovine pancreas, V4129) were used. Both gastric and duodenal digestion were terminated by the addition of trichloroacetic acid (TCA, T6399), according to Bax et al. [17] and Sun et al. [24] with minor modifications. Prior to the simulated gastric and duodenal conditions, all of the chemical reagents and the meat homogenate were pre-heated to 37 °C in a water bath. All chemicals were obtained from Sigma-Aldrich (Steinheim, Germany).

#### 2.6.2. Meat Preparation

Prior to the experiments, whole pork muscles (cooked according to specifications in Table 1) were minced for 10 s (Mini-Kitchen 9440, WIK, Essen, Germany). Subsequently, 2 g of minced meat was suspended in 20 mL of 0.01 M phosphate buffer (pH = 7.4) and homogenized at 20,500 rpm for 30 s (Ultra Turrax T25, Ikka Labortechnik, Staufen, Germany). The protein concentration of each sample was measured by taking separate aliquots at a dilution factor of 1:40 (sample was mixed with 5% sodium dodecyl sulfate (SDS), 8 M Urea, 1 M Dithiothreitol, and the absorbance at 280 nm was subsequently measured using SpectraMax i3x Platform (Molecular Devices, Inc., Danaher Corporation, Sunnyvale, CA, USA). 

#### 2.6.3. Simulated Gastric Conditions

In a 50 mL glass beaker, 10 mL of the diluted meat homogenate was mixed with 7.5 mL of simulated gastric fluid (SGF Electrolyte Stock Solution), 5 µL of 0.3 M CaCl_2_, 0.2 mL of 1 M HCl, and 0.695 µL of Millipore water in a shaking water bath (preheated to 37 °C). The pH was adjusted to pH 3.00. Then, 1.6 mL of gastric pepsin (2000 U/mL, EC 3.4.23.1) was added to reach a final volume of 20 mL. Aliquots of 400 µL of the gastric mixture were transferred to Eppendorf tubes at various time points (0, 10, 20, 30, 60, and 120 min) during gastric digestion. Gastric digestion was terminated by the addition of 800 µL of 20% TCA (1.22 N) to each aliquot while the samples were placed on ice. After centrifugation at 10,000 g for 15 min at 4 °C, 200 µL of the supernatant was pipetted onto a quartz microtiter plate. The hydrolyzed peptide content expressed as optical density (OD) was measured at 280 nm by using SpectraMax i3x Platform (Molecular Devices, Inc., Danaher Corporation, Sunnyvale, CA, USA). 

#### 2.6.4. Simulated Duodenal Conditions

After simulated gastric conditions, 200 µL of 1 M NaOH was added to the meat homogenates to inactivate pepsin activity. In a 50 mL glass beaker, 10 mL of the gastric digest was mixed with 4.25 mL of simulated intestinal fluid (SIF Electrolyte Stock Solution), 1.25 mL of bile solution (160 mM), 20 µL of 0.3 M CaCl_2_, 40 µL of 1 M NaOH, and 0.695 µL of Millipore water in a shaking water bath (37 °C). The pH was adjusted to pH 8.00 with 0.1 M HCL and 0.1 M NaOH. Then 2.5 mL of trypsin (100 U/mL, EC 3.4.21.4) and 1.25 mL of α-chymotrypsin (25 U/mL, EC 3.4.21.1) were added simultaneously to reach a final volume of 20 mL. Aliquots of 400 µL of the duodenal mixture were taken at various time points (0, 10, 20, 30, 60, and 120 min) during duodenal digestion. As with the gastric protocol, digestion was terminated by the addition of 800 µL of 20% TCA, and samples were placed on ice throughout duodenal digestion. After centrifugation at 10,000 g for 15 min at 4 °C, 200 µL of the supernatant was extracted and then measured at 280 nm. All of the analyses were performed in triplicates taken from the same batch of the homogenates. The measured OD values expressed the proteolytic activity, and the rate of proteolysis was calculated as OD units per hour (ΔOD/h).

### 2.7. Statistical Analysis

Statistical analysis was performed using R (version 3.1.2, R Core Team, 2015, www.r-project.org). A significance level of 0.05 was used, while *p* values between 0.1 and 0.05 were regarded as tendencies. A power calculation was performed for ad libitum EI, which was the primary endpoint. The sample size was based on the calculations in Gregersen et al. [25]. According to Gregersen et al. [25], 35 participants must be included in a paired design to detect a difference of 500 kJ in ad libitum EI between the meals, with a statistical power of 0.9 and a significance level of 0.05. The effect of the meal on satiety, hunger, fullness, prospective intake, and the composite satiety score (CSS) was investigated using repeated measurements as well as a summary measure: the incremental area under the curve (iAUC) for satiety, fullness, and CSS, or the incremental area over the curve (iAOC) for hunger and prospective food intake. The iAUC and iAOC were calculated using the trapezoidal method (the sum of the areas of the triangles/trapezoids between each time point adjusted for baseline values). Repeated measurements were analyzed using a linear mixed model ANCOVA, including the meal-time interaction, visit, liking, and baseline as fixed effects and subject, and within-visit subject as random effects. Liking was included in the model, since liking showed a significant difference between the meals. Moreover, residual errors in the repeated measurement models were assumed to be serially correlated within each visit for each subject, following a Gaussian correlation structure with exponentially decreasing correlation with increasing time gaps squared increase. Model checking for variance homogeneity and normal distributions were based on residual plots and normal probability plots. For iAUC/iAOC, ad libitum EI, and ratings of liking, linear mixed models were used including meal, visit, and liking (only in the iAUC/iAOC models) as fixed effects and subjects as random effects. In case of significant main effects of a meal, post-hoc comparisons were performed with the Tukey-Kramer adjustment of significance levels for pairwise comparison. Peak/nadir values and time to peak/nadir were analyzed using a linear mixed model ANOVA with an adjustment for visit number and baseline (only in models analyzing differences in peak/nadir), and including subject as random effects. OD and ΔOD/h were analyzed by repeated measurement using a linear mixed model ANCOVA, including meal-time interaction and baseline (only included in the model for OD) as fixed effects and sample as random effects. A one-way ANOVA and Tukey-Kramer adjustment for pairwise comparison was performed on pH, shear force, cooking loss, maximum degradation (OD_max_), and maximum rate of digestion to detect differences between the meals. For each of our outcomes, four individually pairwise comparisons were performed in order to address our *a priori* hypotheses: (1) LTLT-R-72m vs. OVEN-R and LTLT-R-17h vs. OVEN-R, (2) LTLT-R-17h vs LTLT-R-72m, and (3) LTLT-M-17h vs. LTLT-R-17h. 

## 3. Results

### 3.1. Baseline Characteristics of Participants

During the study, two participants dropped out due to time constraints. Since these two drop-outs had only completed one of the four study days, they were not included in the final data analysis. One participant was excluded due to failure to comply with the study protocol, giving a total of *n* = 37 in the data analysis. The 37 study participants were healthy men: (mean ± standard deviation (SD)) age: 43 ± 9.9 years; body weight: 81.3 ± 8.8 kg; BMI: 24.3 ± 2.3 kg/m^2^. 

### 3.2. Ad Libitum Energy Intake

**Figure 1:**
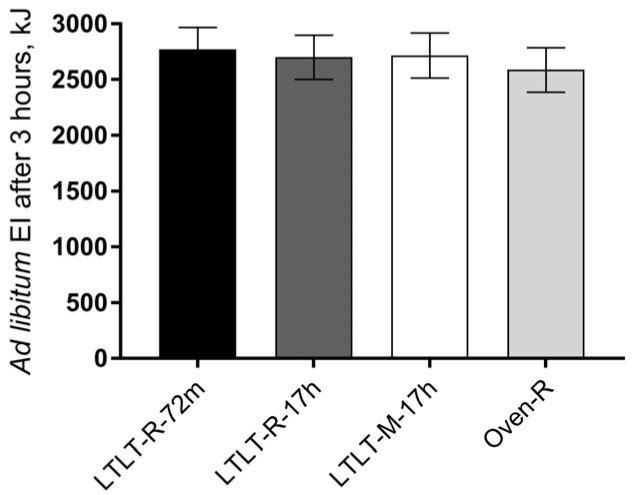
Ad libitum energy intake (mean ± standard error of the mean (SEM), *n* = 37) three hours after the test meals. The test meals were prepared according to Table 1. Repeated measurement analysis, differences between meals, *p* = 0.7.

There were no significant differences in ad libitum EI three hours after the test meal between the four test cooking protocols (*p* = 0.7) (Figure 1).

### 3.3. Subjective Appetite Sensations 

**Figure 2:**
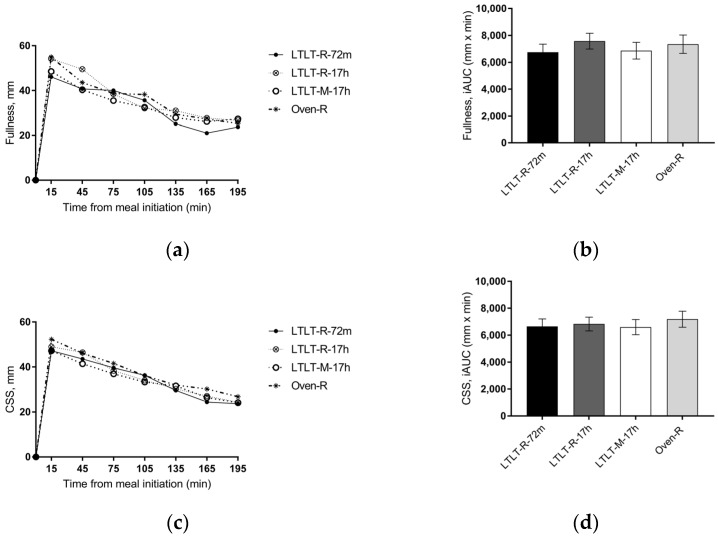
Fullness: (**a**) mean 3-hour response and (**b**) incremental area under the curve (iAUC). Composite satiety score (CSS): (**c**) mean 3-hour response, and (**d**) corresponding iAUC. Values are means ± SEM, *n* = 37. The test meals were prepared according to Table 1.

No interaction between time and treatment was observed for any of the subjective appetite ratings. Postprandial ratings of fullness differed between the meals when analyzed as repeated measures (*p* < 0.05) (Figure 2). The OVEN-R meal tended to increase fullness when compared to the LTLT-R-72m (*p* = 0.07) and LTLT-M-17h (*p* = 0.08) meals. Moreover, peak level of sensation of fullness was higher after the OVEN-R meal as compared to the LTLT-R-72m meal (*p* < 0.01). There were, however, no differences when fullness was summarized as iAUC (*p* = 0.42). For prospective food intake, no overall meal effect was observed in the postprandial response or in iAOC. However, the nadir level was lower for the OVEN-R meal as compared to the LTLT-R-72m meal (*p* < 0.05). Postprandial ratings of satiety and hunger and their related summary measures did not differ between the meals, *p* > 0.05. Also, no difference in peak or nadir levels for satiety and hunger was observed. The composite satiety score (CSS) combined the four appetite ratings into one score (Figure 2). There was a tendency towards an overall meal effect (*p* = 0.05), with the OVEN-R meal tending to increase CSS as compared to the LTLT-R-72m (*p* = 0.06). The peak level of CSS was higher after the OVEN-R meal as compared to the LTLT-R-72m meal (*p* < 0.05). 

### 3.4. Palatability

**Figure 3:**
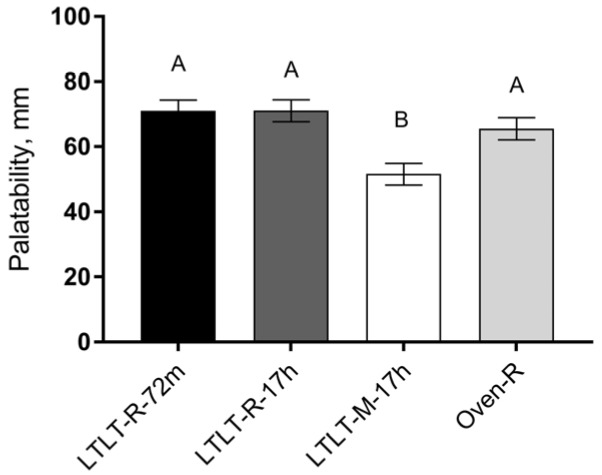
Palatability of the test meals measured on a 100 mm line scale from “Not at all like” to “Like a lot”. (LS means ± SEM, *n* = 37)). The test meals were prepared according to Table 1. Different letters for each bar indicate a statistical difference (*p* < 0.05).

The palatability of the meal containing LTLT-cooked minced pork patties (LTLT-M-17h) was rated significantly lower than that of the three other meals containing roast (*p* < 0.001) (Figure 3). No differences in palatability were observed between the meals containing roast. 

### 3.5. Physical and In Vitro Digestion Parameters of Pork

The shear force of cooked *Semitendinosus* was affected by the holding time (*p* < 0.05) (Table 2). A holding time of 17 h during LTLT cooking reduced toughness significantly as compared to 72 min (*p* < 0.05) and oven cooking at 160 °C (*p* < 0.05). However, the shorter LTLT holding time of 72 min did not decrease toughness compared to oven cooking (*p* = 0.99). 

**Figure 4:**
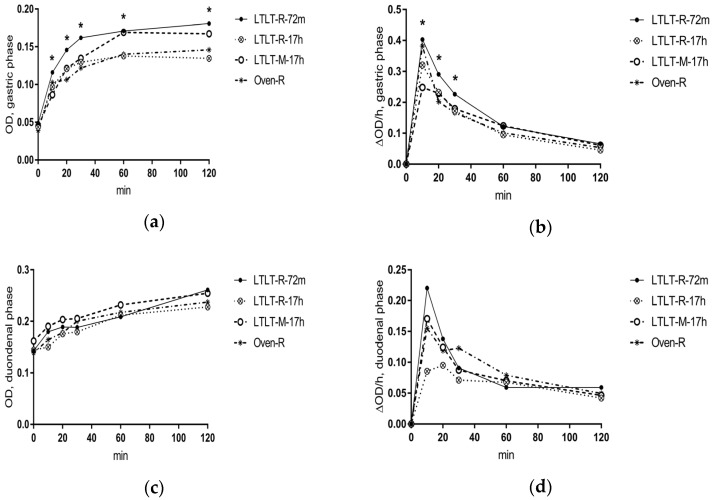
In vitro protein digestion in gastric phase: (**a**) optical density (OD) expressing the proteolytic activity and (**b**) the rate of proteolysis (ΔOD/h) and in the duodenal phase: (**c**) OD, and (**d**) ΔOD/h. Significant differences at specific time points are indicated by *. The test meals were prepared according to Table 1.

Results of the in vitro digestion parameters of the meat are presented in Figure 4 and Table 2. OD and ΔOD/h values expressed the proteolytic activity and rate of proteolysis, respectively. In the gastric phase, a time-meal interaction was detected for OD and ΔOD/h (*p* < 0.001) (Figure 4), indicating that differences between the meals varied over time. LTLT-R-72m pork had higher OD values than LTLT-R-17h pork at all time points (*p* < 0.01), except at time points 10 and 40 min, where no significant differences were found. Also, the LTLT-R-72m pork had higher OD values than pork in the OVEN-R meal in the early digestion phase (20–30 min) (*p* < 0.05). In the late digestion phase (120 min), the LTLT-M-17h pork and OVEN-R pork resulted in higher OD values than the LTLT-R-17h pork (*p* ≤ 0.01). The rate of proteolysis (ΔOD/h) was higher in the LTLT-R-72m pork than in the LTLT-R-17h pork in the early gastric digestion phase (10–30 min) (*p* < 0.05), but not in the late phase (40–120 min). Also, the LTLT-72m pork had higher ΔOD/h values than the OVEN-R pork; however, significant differences were only found at time points 20 and 30 min (*p* < 0.05). 

In the intestinal digestion phase, no time-meal interaction was observed for the in vitro digestion parameters. There was a significant meal effect for OD (*p* < 0.05), but not for ΔOD/h (*p* = 0.13) (Figure 4). Pairwise comparisons showed that the LTLT-R-72m pork had higher OD values than the LTLT-R-17h pork (*p* < 0.05). Moreover, the LTLT-M-17h pork resulted in higher OD values than the LTLT-R-17h pork (*p* < 0.01).

## 4. Discussion

Our results showed that the cooking method (LTLT cooking compared to oven cooking), the LTLT holding time (72 min compared to 17 h), and the pork structure (minced pork as compared to pork roast) did not have a clear effect on ad libitum EI nor subjective appetite sensations, although small differences were found for some of the outcomes on subjective appetite. Results from in vitro digestion showed that the cooking method and the LTLT holding time had an effect on the proteolytic rate in the gastric phase, whereas the pork structure did not consistently affect digestion parameters at all. The proteolytic rate was higher in the early gastric phase for LTLT cooking for 72 min, as compared to LTLT cooking for 17 h or oven cooking, but with no differences in the intestinal phase. 

Our primary endpoint, ad libitum EI, was not affected differently by the four cooking protocols, and we were therefore not able to confirm any of our hypotheses. This was supported by our results on subjective appetite ratings of satiety, fullness, hunger, and prospective food intake, which did not differ clearly between the four cooking protocols. Thus, changes in eating behavior (i.e., food intake) were expected to arise from changes in sensations of hunger or satiety. To our knowledge, this is the first study to investigate the effect of LTLT cooking on the satiating properties of dietary protein and possible underlying mechanisms. Only one trial has compared different cooking techniques of beef on appetite [26], although the meals in the beef study were not designed to be isocaloric or macronutrient-matched. Dietary protein is one of the most satiating macronutrients [2], and seems to increase satiety in a dose-response manner [4]. Different mechanisms related to post-ingestive and post-absorptive signals have been proposed for the satiating effect of protein [27,28]. Our hypotheses were based on the aminostatic hypothesis [5], which has been suggested as one of the underlying mechanisms of protein-induced satiety [8,27]. Further, increased concentrations of plasma amino acids resulting from rapidly digested meat proteins would induce satiety [6]. 

Recently published studies have, however, shown that the ingestion of bioaccessible proteins does not induce satiety per se. Bendtsen et al. [29] investigated three high-protein meals based on hydrolyzed casein, intact casein, and intact whey in 24 overweight and moderately obese young men and women. Although whey is considered to be a rapidly digested protein and casein a more slowly digested protein [30], no differences were observed in appetite regulation or energy expenditure between the meals. Similar results were found in a long-term cross-over study that compared two weight-loss diets containing protein (30 E%), where one of the diets contained a mixture of protein and free amino acids (each 15 E% of EI) [31]. The aminostatic hypothesis has therefore recently been challenged, which is also supported by our study. 

Several reasons could explain why the four test meals based on different meat cooking protocols induced similar effects on appetite. Firstly, the meat was ingested as part of whole meals containing other foods (rice and sauce), thereby providing other nutrients than proteins. However, fat and dietary fiber have been shown to reduce the gastrointestinal transit time [32], which might decrease protein digestibility and thus shield potential satiating effects from meat proteins. 

Secondly, the meat proteins may have undergone different modifications during cooking than expected. The initial OD values in gastric digestion expressed the amount of endogenously hydrolyzed peptides and amino acids present in the meat prior to gastric digestion (Table 2). Christensen et al. [11] have shown that LTLT-cooked pork cooked at 58 °C and with increasing holding times had higher proteolytic activity from endogenous cathepsins B + L than pork cooked at higher temperatures. It was therefore expected that LTLT-cooked pork would contain a higher concentration of peptides and free amino acids. However, we did not find differences in the initial OD values between any of the meals. Therefore, contrary to our expectations, it does not seem that LTLT cooking increases the levels of endogenously hydrolyzed peptides and amino acids. 

However, the results from in vitro simulated digestion showed that the cooking method and LTLT holding time affected in vitro protein digestibility. Both in vitro and in vivo experiments in animals have shown that the cooking temperature modulates the protein digestion rate [17,33]. Cooking at 70 °C as compared to 100 °C or above has been shown to increase protein digestibility due to denaturation, thereby increasing the accessibility of cleavage sites to gastrointestinal enzymes [17]. On the other hand, cooking at high temperatures [17] or for a prolonged time [34] could result in protein-protein interaction, leading to aggregation. Protein aggregation limits the accessibility to enzymes during digestion and may therefore explain the slower proteolytic rate of the oven-cooked pork during in vitro digestion.

As expected, the shear force decreased with increased holding times, indicating that structural modifications of meat components and proteins had occurred. We also found that the proteolytic rate of pepsin in the early in vitro digestion decreased with increased holding times (17 h vs. 72 min). During cooking, meat proteins undergo oxidative modifications, and a long cooking time of up to 24 hours has been shown to increase protein carbonylation in LTLT-cooked lamb loins independently of cooking temperature [34]. Furthermore, during cooking, protein aggregation from increased protein surface hydrophobicity may occur [35]. These physico-chemical changes in the meat proteins could decrease proteolytic accessibility to pepsin [35], and thus decrease protein digestibility. The present results were inconsistent with our hypothesis that an increased holding time in LTLT cooking would increase the protein digestion rate. However, different processes with antagonistic effects on protein digestibility seem to co-exist during cooking: aggregation, protein unfolding, and endogenous enzymatic cleavage. For 17-hour cooking, aggregation could have reduced the accessibility to cleavage active sites of unfolded proteins, and thus counteracted the enzymatic activity, leading to a generally lower digestion rate. 

Mincing of pork did not have an effect on the in vitro proteolytic rate. Minced beef has been shown to be more rapidly digested and absorbed than beef steak in an in vivo study. This was concluded in a cross-over study with ten elderly men, who consumed intrinsically labelled beef in order to study protein metabolism [19]. Both minced beef and beef steak were grilled until a core temperature of 65 °C was reached. In our study, minced pork and pork roast were LTLT cooked at 58 °C for a holding time of 17 h. This long holding time may have caused protein aggregation, as discussed above, and might have counteracted any possible positive effects of the mincing on protein digestibility. 

In general, digestibility of meat proteins is high, and almost all amino acids are absorbed [8,9]. However, the rate of digestion could be of interest in relation to satiety. Proteins that undergo faster digestion and absorption could modify the aminoacidemia response, which potentially induces a faster satiety response [5,6]. The present results showed that LTLT cooking for a holding time of 72 min increased the proteolytic rate in early gastric digestion, when compared to oven cooking. However, these effects did not persist in the intestinal phase. Since no physiological markers of protein digestion or absorption were included in the present study, any conclusions on *in vivo* effects are limited. 

The strengths and limitations of the present study should be considered. We consider it a strength that we used a whole meal approach with realistic foods, since such meals would resemble the kind of food consumed outside the experimental setting. In our design, we aimed to compare two cooking methods in relation to satiety and in vitro protein digestibility. Convection oven cooking was chosen as a comparator to LTLT cooking, where pork in each of the meals had a core temperature of 58 °C. However, in oven cooking, the oven temperature was 160 °C, resulting in a temperature gradient [36] across the roast ranging from 160 °C on the surface to 58 °C in the core. In LTLT cooking, a uniform heat treatment of 58 °C was achieved. The physical and chemical processes of meat proteins are highly dependent on heating [13,17], and therefore the oven cooking might have resulted in a mixture of modifications to the meat proteins. Thus, oven cooking may not be an appropriate comparator to the precisely controlled temperatures in LTLT cooking. However, we chose oven cooking, since this is how meat is typically heated. Lastly, panfrying of the meat prior to serving may have induced protein changes on the surface of the meat, which might have blurred the positive effect of LTLT cooking. Nevertheless, it was decided to mimic a realistic meal.

## 5. Conclusions

In conclusion, meals based on pork with differences in the cooking method (LTLT cooking vs. oven cooking), the LTLT holding time (17 h vs. 72 min), or the pork structure (minced pork vs. pork roast) induced similar effects on ad libitum EI and subjective appetite ratings. We also conclude that the *in vitro* gastric digestion of meat proteins was faster after LTLT at 58 °C for 72 min as compared to oven cooking at 160 °C and a longer LTLT holding time of 17 hours. Future studies investigating the effects of cooking methods on meat protein digestibility should include additional analytical measures to obtain a better understanding of the structural and chemical changes. 

## Figures and Tables

**Table 1:** Nutritional composition of the four test meals in the meal test study.

**Nutritional Composition**	**LTLT-R-72m Meal**	**LTLT-R-17h Meal**	**LTLT-M-17h Meal**	**OVEN-R Meal**
	Roast pork (202 g)	Roast pork (202 g)	2 pork patties (179 g) ^1^	Roast pork (202 g)
	225 g rice	225 g rice	225 g rice	225 g rice
	75 mL sauce	75 mL sauce	75 mL sauce	75 mL sauce
	220 mL water	220 mL water	220 mL water	220 mL water
Meal weight, g	722	722	699	722
Energy, kJ	2813	2919	2915	2936
Fat, g	20	21	21	22
E%	27	27	27	27
Protein, g	57	61	61	62
E%	34	36	36	36
Carbohydrates, g	63	63	63	63
E%	38	37	37	37
Dietary fiber, g	2	2	2	2

^1^ Adjusted for cooking loss as described in Table 2. LTLT: Low temperature long time cooking; E%: energy percent; kJ: kilo joule, LTLT-R-72m: LTLT-cooked roast at 58 °C for 72 min, LTLT-R-17h: LTLT-cooked roast at 58 °C for 17 h, LTLT-M-17h: LTLT-cooked minced pork patties at 58 °C for 72 min, OVEN-R: oven-cooked roast at 160 °C until 58 °C in core.

**Table 2:** Physical and in vitro digestion parameters of cooked porcine *M. Semitendinosus* used in the appetite study (mean ± SEM unless otherwise specified).

Physical and In Vitro Digestion Parameters	LTLT-R-72m Pork	LTLT-R-17h Pork	LTLT-M-17h Patties	OVEN-R Pork	*p*
Shear force (N) ^1^	43.9 ± 3.6 ^a^	32.7 ± 6.7 ^b^	-	43.4 ± 6.5 ^a^	0.02
Cooking loss (%) ^2^	21.6 ± 0.8 ^a^	27.6 ± 0.3 ^b^	35.7 ± 0.28^c^	28.5 ± 0.3 ^b^	<0.001
**Pepsin digestion** ^3^					
- Initial OD	0.05 ± 0.01	0.04 ± 0.01	0.05 ± 0.01	0.04 ± 0.00	0.56
- OD_max_	0.18 ± 0.01	0.14 ± 0.02	0.17 ± 0.01	0.15 ± 0.01	0.13
- ΔOD/h_max_	0.40 ± 0.01	0.32 ± 0.06	0.25 ± 0.04	0.38 ± 0.02	0.06
- Time of ΔOD/h_max_ (min)	10	10	10	10	0.44
**Duodenal digestion** ^3^					
- Initial OD	0.14 ± 0.00	0.14 ± 0.01	0.16 ± 0.01	0.14 ± 0.01	0.18
- ODmax	0.26 ± 0.01	0.23 ± 0.01	0.25 ± 0.00	0.24 ± 0.01	0.09
- ΔOD/hmax	0.24 ± 0.07	0.12 ± 0.04	0.19 ± 0.05	0.17 ± 0.02	0.32
- Time of ΔOD/hmax (min)	15 ± 5.0	20 ± 5.8	13.3 ± 3.3	20 ± 5.8	0.72

Different letters for each column indicate a statistical difference (*p* < 0.05). ^1^ mean ± SD, *n* = 5. ^2^ cooking loss from LTLT/oven cooking and pan-frying (whole muscles: *n* = 51; meat patties: *n* = 50). ^3^ *n* = 3. OD: optical density expressing the proteolytic activity, ΔOD/h_max_: the maximal rate of proteolysis, LTLT-R-72m: LTLT-cooked roast at 58 °C for 72 min, LTLT-R-17h: LTLT-cooked roast at 58 °C for 17 h, LTLT-M-17h: LTLT-cooked minced pork patties at 58 °C for 72 min, OVEN-R: oven-cooked roast at 160 °C until 58 °C in core.
